# Modelling Complex Bimolecular Reactions in a Condensed Phase: The Case of Phosphodiester Hydrolysis

**DOI:** 10.3390/molecules28052152

**Published:** 2023-02-24

**Authors:** Alessandro Nicola Nardi, Alessio Olivieri, Andrea Amadei, Riccardo Salvio, Marco D’Abramo

**Affiliations:** 1 Dipartimento di Chimica, Università degli Studi di Roma La Sapienza, P. le Aldo Moro 5, 00185 Roma, Italy; 2 Dipartimento di Scienze e Tecnologie Chimiche, Università degli Studi di Roma Tor Vergata, Via della Ricerca Scientifica 1, 00133 Roma, Italy; 3 ISB—CNR Sezione Meccanismi di Reazione, Università degli Studi di Roma La Sapienza, 00185 Roma, Italy

**Keywords:** phosphodiester hydrolysis, PMM method, molecular dynamics, rate constant calculation, statistical mechanics, quantum chemistry

## Abstract

(1) Background: the theoretical modelling of reactions occurring in liquid phase is a research line of primary importance both in theoretical–computational chemistry and in the context of organic and biological chemistry. Here we present the modelling of the kinetics of the hydroxide-promoted hydrolysis of phosphoric diesters. (2) Method: the theoretical–computational procedure involves a hybrid quantum/classical approach based on the perturbed matrix method (PMM) in conjunction with molecular mechanics. (3) Results: the presented study reproduces the experimental data both in the rate constants and in the mechanistic aspects (C–O bond vs. O–P bond reactivity). The study suggests that the basic hydrolysis of phosphodiesters occurs through a concerted $A_{N}D_{N}$ mechanism, with no formation of penta-coordinated species as reaction intermediates. (4) Conclusions: the presented approach, despite the approximations, is potentially applicable to a large number of bimolecular transformations in solution and therefore leads the way to a fast and general method to predict the rate constants and reactivities/selectivities in complex environments.

## 1. Introduction

Phosphodiester bonds are the linkages that connect biological information stored in nucleic acids. Their resistance to hydrolysis [1] makes them ideal for preserving biochemical information. However, phosphodiester hydrolysis is a crucial process in biochemical systems and for this reason, nature has developed a number of enzymes able to catalyse the hydrolysis of these compounds in aqueous solution. The rate enhancements induced by these enzymes (e.g., kinases, ATPases, phosphatases) are astonishing (up to $10^{17}$ in the case of *staphylococcal nuclease*) [2,3]. On the other hand, numerous research groups have designed and synthesized small molecule enzyme mimics able to cleave DNA, RNA and their model compounds in an effective and selective fashion [4,5,6,7].

The mechanism of phosphodiester hydrolysis was investigated at different pH conditions and for diverse phosphodiesters through experimental and theoretical–computational approaches for both catalysed [2,4,8,9,10,11,12,13,14] and uncatalysed reactions [9,15,16,17,18]. Both approaches present some critical aspects. The measurements of the rate constants for the uncatalysed hydrolysis of phosphodiesters are hampered by the extremely long half-life time of the reaction (e.g., 30,000,000 years at 25 °C) [2]. For this reason, there is the need to carry out kinetic experiments at elevated temperatures and pressures and obtain the reaction rate at room temperature by extrapolation [2]. Regarding the computational approach, the problems manifest in the choice of the proper method to describe the reaction coordinate and the solvent effect. For the reasons illustrated above, the development of an efficient and reliable method to investigate phosphodiester hydrolysis is crucial to provide useful insights into the mechanisms of these reactions and to evaluate the catalytic enhancement offered by the enzymes and artificial catalysts with respect to spontaneous hydrolysis.

In this work, we focused on the study of the hydrolysis of a simple phosphodiester, dineopentyl phosphate, hereafter referred to as $Np_{2}P^{-}$. The hydrolysis of this substrate was investigated by Wolfenden et al. through an in-depth kinetic analysis [2], and therefore experimental data are available for a direct comparison. This compound has the tendency, similarly to DNA, to undergo cleavage of the phosphorous–oxygen bond, instead of the carbon–oxygen bond as many phosphoric diesters [2]. This reactive behaviour can be attributed to the steric hindrance provided by the *t*-butyl units that force the attack of the upcoming nucleophile to the phosphorous atom, as illustrated and discussed in detail in the present manuscript (*vide infra*. Experimental data indicate that the hydroxide-catalyzed hydrolysis dominates over the spontaneous reaction as the leaving groups become poorer [2] and, according to this evidence, we have selected the hydroxide ion attack onto the substrate, instead of a water molecule, as a test reaction. The hydroxide attack is also frequent in enzyme-catalysed reactions, where a metal cation helps the deprotonation of a water molecule [8,19,20]. This transformation was modelled by a statistical-mechanical approach based on a quantum mechanics/molecular mechanics hybrid method. The results obtained by this approach are illustrated and discussed in detail and compared with the experimental data.

## 2. Theory

### 2.1. General Considerations

Let us consider a simple reaction from the reactant (*R*) chemical state to the product (*P*) chemical state, without involving any distinguishable intermediate chemical state, as occurring within a single reactive centre (RC), either a molecule or a complex, interacting with its environment. Note that we assume for the *R* and *P* chemical states a fully in equilibrium: i.e., the corresponding mean residence time is large enough to allow for internal relaxation. We can also reasonably assume that the reaction events must pass trough a specific and unique transition state (TS) with a mean traversing time too short for any internal relaxation, hence providing the reaction scheme

(1) $$R\overset{k_{R}}{\to }TS\overset{k}{\to }P$$

and thus

(2) $$\begin{array}{ccc} \dot{\left[R\right]} & = & -k_{R}\left[R\right] \end{array}$$

(3) $$\begin{array}{ccc} \dot{\left[TS\right]} & = & k_{R}\left[R\right]-k\left[TS\right] \end{array}$$

(4) $$\begin{array}{ccc} \dot{\left[P\right]} & = & k[TS] \end{array}$$

Considering that we necessarily have $k\gg k_{R}$ we can assume the steady state for TS (i.e., $\dot{\left[TS\right]}≅0$) providing

(5) $$\begin{array}{ccc} \left[TS\right] & ≅ & \frac{k_{R}}{k}\left[R\right] \end{array}$$

(6) $$\begin{array}{ccc} \dot{\left[P\right]} & ≅ & k\frac{k_{R}}{k}\left[R\right]=k_{R}\left[R\right] \end{array}$$

It is reasonable to assume that within the TS equilibrium ensemble the outward flux rate constants are identical, given by $k_{TS}≅k/2$ (only one half of the TS population is moving towards *P*), and thus from Equation (6) we have

(7) $$\begin{array}{ccc} \dot{\left[P\right]} & ≅ & -\dot{\left[R\right]}≅\frac{k_{R}}{k_{TS}}\;\frac{k}{2}\left[R\right]=\frac{Q_{TS}}{Q_{R}}\;\frac{k}{2}\left[R\right]\\ & = & K_{R}\left[R\right] \end{array}$$

with $Q_{TS}$ and $Q_{R}$ the canonical partition functions of the whole system with a selected single RC complex (the reference RC) constrained in the TS or the *R* state, respectively (note that for reactive centres at infinite dilution the whole system can be considered as including a single RC embedded into a huge amount of solvent molecules). From Equation (7) it readily follows that the reaction process can be described by the simplified reaction scheme

(8) $$R\overset{K_{R}}{\to }P$$

### 2.2. The Reaction Coordinate, the Landau Free Energy and the Rate Constant

**Figure 1:**
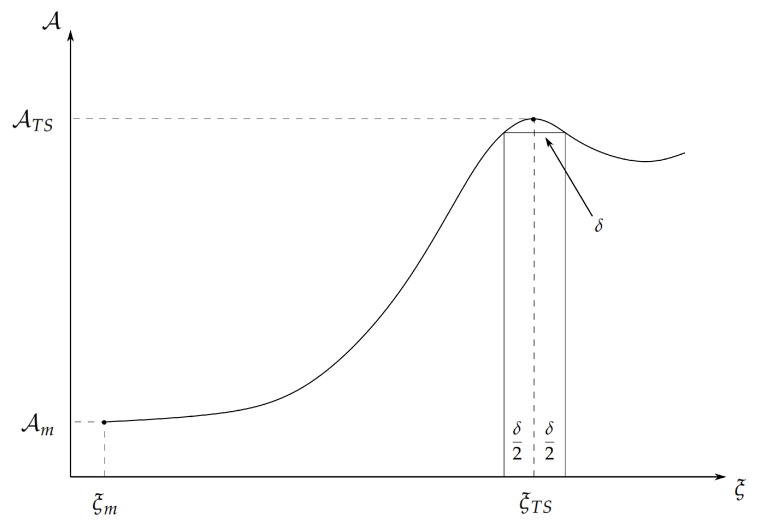
Pictorial representation of a generic free energy profile along the reaction coordinate ξ.

Let us assume that we can identify a generalized coordinate ξ (i.e., the reaction coordinate) univocally defining, via its different intervals, the *R*, TS and *P* chemical states of the reference RC (infinite dilution at the reactive centre the single RC is present), and thus properly describing the reaction process (see Figure 1). Defining with $\left[\xi_{TS}-\delta /2,\xi_{TS}+\delta /2\right]$ the TS tiny reaction coordinate range and with $\xi_{m}$ the minimum ξ value of the *R* chemical state, we can express the partition functions $Q_{R}$ and $Q_{TS}$ via the Landau free energy A defined by

(9) $$A\left(\xi \right)=-k_{B}T\ln Q\left(\xi \right)$$

with Q(ξ) the partition function density providing

(10) $$\begin{array}{ccc} Q_{R} & = & \int_{\xi_{m}}^{\xi_{TS}-\delta /2}Q\left(\xi \right)d\xi =\int_{\xi_{m}}^{\xi_{TS}-\delta /2}e^{-\beta A(\xi )}d\xi ≅\int_{\xi_{m}}^{\xi_{TS}}e^{-\beta A(\xi )}d\xi \end{array}$$

(11) $$\begin{array}{ccc} Q_{TS} & = & \int_{\xi_{TS}-\delta /2}^{\xi_{TS}+\delta /2}Q\left(\xi \right)d\xi =\int_{\xi_{TS}-\delta /2}^{\xi_{TS}+\delta /2}e^{-\beta A(\xi )}d\xi ≅e^{-\beta A(\xi_{TS})}\delta \end{array}$$

where $1/\beta =k_{B}T$ and we used $A\left(\xi \right)≅A\left(\xi_{TS}\right)$ within the TS range.

From Equations (7), (10) and (11) we obtain

(12) $$\begin{array}{ccc} K_{R} & ≅ & \frac{e^{-\beta \Delta A_{R}^{†}}\delta }{\int_{\xi_{m}}^{\xi_{TS}}e^{-\beta \Delta A(\xi )}d\xi }\;\frac{k}{2} \end{array}$$

(13) $$\begin{array}{ccc} \Delta A(\xi ) & = & A\left(\xi \right)-A\left(\xi_{R}\right) \end{array}$$

(14) $$\begin{array}{ccc} \Delta A_{R}^{†} & = & A\left(\xi_{TS}\right)-A\left(\xi_{R}\right) \end{array}$$

where $\xi_{R}$ is a reference position within the *R* state range (typically the Landau free energy minimum). Realizing that the TS, corresponding to a tiny ξ interval, is characterized by a constant density within the whole δ range and for each reaction event by a virtually constant dξ/dt velocity, we can express the outward flux (forming the *P* population) via k[TS]≅[TS]〈vs.〉/δ with 〈vs.〉 the mean TS traversing velocity given by the equilibrium mean value of v=|dξ/dt|. Therefore, it follows

(15) $$k≅\frac{\langle vs.\rangle }{\delta }$$

readily providing

(16) $$K_{R}≅\frac{e^{-\beta \Delta A_{R}^{†}}}{\int_{\xi_{m}}^{\xi_{TS}}e^{-\beta \Delta A(\xi )}d\xi }\;\frac{\langle vs.\rangle }{2}$$

Finally, we can obtain the Landau free energy change along ξ, via [21]

(17) $$\begin{array}{ccc} A\left(\xi \right)-A\left(\xi_{ref}\right) & = & A_{\xi }-A_{\xi_{ref}}≅-k_{B}T\ln{\langle e^{-\beta \Delta U}\rangle }_{\xi_{ref}} \end{array}$$

(18) $$\begin{array}{ccc} A_{\xi } & = & A\left(\xi \right)-k_{B}T\ln\delta \end{array}$$

(19) $$\begin{array}{ccc} A_{\xi_{ref}} & = & A\left(\xi_{ref}\right)-k_{B}T\ln\delta \end{array}$$

(20) $$\begin{array}{ccc} \Delta U & = & U\left(\xi \right)-U\left(\xi_{ref}\right) \end{array}$$

where the subscript of the angle brackets indicate averaging within the $\xi_{ref}$ reference ensemble, U(ξ) and $U(\xi_{ref})$ are the perturbed electronic energies (estimated by means of the perturbed matrix method (PMM) [22,23]) as obtained either at fixed ξ or at fixed $\xi_{ref}$ for each accessible semi-classical configuration, once relaxed all the quantum vibrational coordinates are at their minimum energy position (we consider the quantum vibrational energy as independent of the ξ coordinate), and $A_{\xi }$ and $A_{\xi_{ref}}$ are the Helmholtz free energies when confining the RC within the tiny δ range either around ξ or around $\xi_{ref}$. Note that in practice Equation (17) is accurate only when ξ is not too far from $\xi_{ref}$, thus allowing us to use the fixed $\xi_{ref}$ MD simulation to reconstruct the ξ ensemble (i.e., the MD simulation provides a reasonable sampling of the relevant phase space regions of both ensembles). Therefore, for evaluating the complete free energy profile we can use a set of reference positions $\xi_{ref,n},n=1,N$ providing a set of $\left[\xi_{ref,n},\xi_{ref;n+1}\right]$ sub-ranges each to be used to obtain the corresponding (inner) Landau free energy changes. By summing the free energy variations over such sub-ranges the complete Landau free energy profile can be reconstructed and hence by averaging over the free energy profiles as obtained by both the $\xi_{ref,1}\to \xi_{ref,N}$ and $\xi_{ref,N}\to \xi_{ref,1}$ calculations we can obtain a reliable estimate of the reaction Landau free energy surface.

### 2.3. Application to the $Np_{2}P$ Hydrolysis Reaction

The general scheme for the hydrolysis reaction considered is

(21) $$Np_{2}P^{-}+HO^{-}\;\overset{k_{A}}{\underset{k_{2}}{\underset{↽}{⇀}}}\;C_{NR}\;\overset{k_{-1}}{\underset{k_{1}}{\underset{↽}{⇀}}}\;C_{R}\;\overset{K_{R}}{\to }\;P$$

where $Np_{2}P^{-}$ is the dineopentyl phosphate, $HO^{-}$ is the hydroxide ion and $C_{NR},C_{R}$ are the non-reactive and reactive complexes, respectively: the former defined by the interacting partners still insufficiently close to alter their covalent structure while the latter corresponding to closely interacting partners possibly undergoing covalent rearrangement (i.e., the *R* state of Scheme (8)). In the previous reaction scheme the association rate constant $k_{A}$ can be obtained via [24,25]

(22) $$k_{A}=4\pi \left(D_{Np_{2}P^{-}}+D_{HO^{-}}\right)r_{0}N_{A}$$

where $r_{0}$ is the maximum distance between $Np_{2}P^{-}$ and $HO^{-}$ within the non-reactive complex (i.e., the radius length of the sphere defining the overall complex $C=C_{NR}+C_{R}$), $D_{Np_{2}P^{-}}$ and $D_{HO^{-}}$ are the free diffusion coefficients of the two reactant molecules characterizing their behaviour for $r\geq r_{0}$, and $N_{A}$ is the Avogadro number. Finally, assuming steady state for $C_{NR}$ and $C_{R}$, we have [25]

(23) $$\dot{\left[Np_{2}P^{-}\right]}≅-k_{H}\left[Np_{2}P^{-}\right]\left[HO^{-}\right]$$

with

(24) $$k_{H}=\frac{K_{R}k_{A}k_{-1}}{\left(k_{2}+k_{-1}\right)\left(k_{1}+K_{R}\right)-k_{1}k_{-1}}≅\frac{k_{A}}{k_{2}}\;\frac{k_{-1}}{k_{1}}\;K_{R}$$

as the overall (hydrolysis) rate constant (note that in Equation (24) we used $k_{1}+K_{R}≅k_{1}$ as following from $K_{R}\ll k_{1}$, see Table 1). By means of proper MD simulations, the PMM approach and the relations described in the theory section we obtained all the kinetic constants to be used in Equation (24).

## 3. Methods

### 3.1. Quantum Chemical Calculations

The quantum chemical calculations were conducted using Orca software package [26]. To model the hydrolysis of dineopentyl phosphate ($Np_{2}P^{-}$) with the attack on the phosphorous atom, we selected as the quantum centre (QC) the dimethyl-substituted phosphate ($DMP^{-}$) and the hydroxide ion. The quantum chemical calculations were performed using density functional theory (DFT) to obtain the intrinsic reaction coordinate (IRC). The IRC calculation requires a guess of the transition state (TS) of the reaction. The initial guess for the transition state structure was taken from previous work [8] and then optimized for a saddle point at the DFT/PBE0 [27] level of theory using the 6-311++G(2d,2p) [28,29] basis set. The analysis of the transition state hessian confirmed that the obtained structure corresponds to a saddle point of the potential energy surface (PES).

The choice of the PBE0 functional was motivated by a previous benchmark work present in the literature [30].

**Figure 2:**
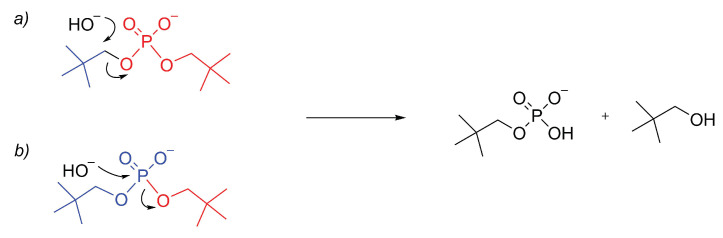
Schematic representation of the alternative reaction mechanisms for the cleavage of $Np_{2}P^{-}$: (**a**) C–O cleavage, $S_{N}2$; and (**b**) P–O cleavage, $S_{N}2$(P) or $A_{N}D_{N}$.

To show that the attack of the hydroxide ion on the neopentyl group is energetically precluded, we performed a PES scan along the hydroxide oxygen atom–dineopentyl carbon atom and the dineopentyl carbon atom–leaving group (see Figure 2a) distances, at DFT/PBE0 [27] level of theory using the 6-31+G(d,p) [28,29,31] basis set. The Cartesian coordinates of the transition states found and used in the present work are available in the Supplementary Materials.

### 3.2. Molecular Dynamics Simulations

The molecular dynamics (MD) simulations were conducted starting from the structures obtained from the IRC calculation (as explained in the previous subsection) and by replacing one hydrogen atom of each methyl group of the $DMP^{-}$ with a *t*-butyl group to obtain a model of the dineopentyl phosphate and hydroxide ion, along the reaction coordinate.

All the molecular dynamics simulations have been performed using Gromacs 2022 software package [32]. The initial structure was centred in a cubic box and solvated using the SPC [33] water model, whereas the force field parameters of the neopentyl phosphate were obtained by means of the ACPYPE package [34]. Two ${\mathrm{Na}}^{+}$ ions have been inserted in the simulation box to reach the electro-neutrality. An energy minimization step was performed using the steepest descent algorithm freezing the atoms corresponding to the QC. After the minimization, a series of equilibration steps, lasting 50 ps with a timestep of 2 fs, were performed in the NVT ensemble: after each run, we checked the density of the system and tune the size of the box in order to reach the correct value of density. After the equilibration steps, each system has been simulated for 25 ns at fixed temperature and volume. The temperature was kept constant at 300 K using the V-rescale thermostat [35] using a $\tau_{T}$ of 0.1 ps. The electrostatic interactions were calculated using the particle mesh Ewald method [36,37] with a cut-off of 1.2 nm. A cut-off of 1.2 nm was used for the van der Waals interactions.

Because the reaction shows a relevant reorganization of the internal degrees of freedom, we divided the intrinsic reaction coordinate into five subparts. For each of them, an MD simulation (maintaining the QC fixed, in a reference structure for the selected subpart) was carried out to provide a proper semi-classical perturbation of the QC.

Additionally, we performed an MD simulation, lasting 100 ns, of the free reactants using a timestep of 2 fs to obtain the hydroxide ion–dineopentyl phosphate distance probability distribution.

A short simulation, lasting 200 ps, with a timestep of 1 fs of the dineopentyl phosphate in aqueous solution was performed to estimate its diffusion coefficient according to the Einstein Equation [38]. For the hydroxide ion, we used the experimental diffusion coefficient reported in [39]. The diffusion coefficients are required for the calculation of the kinetic constant $k_{A}$ (see Scheme (21)) according to Equation (22).

We also performed a set of 70 simulations, lasting 500 ps, starting from random initial configuration within the hydroxide ion–dineopentyl phosphate distance of 0.5–0.6 nm to reconstruct the reactive and non-reactive complex dissociation kinetics.

### 3.3. Computational Strategy

The computational strategy can be summarized in the following steps:

- Calculation of the unperturbed quantum chemical properties, needed to apply the MD-PMM procedure, for each point along the reaction coordinate (electronic ground state energy, permanent dipole moments and CHELPG charges [40]).
- Simulations of selected representative ensemble along the reaction coordinate (reactants, two intermediate structures between reactants and transition state, corresponding to different nucleophile–phosphorous atom distances, and the transition state) providing the perturbation along the reaction coordinate.
- MD-PMM calculation for each point to obtain the free energy difference from one point to the following along the reaction coordinate, according to Equation (17).
- Calculation of the kinetic rate constants involved in the reactive non-reactive equilibrium in Scheme (21) by performing different sets of MD simulations: (*i*) a 100 ns long simulation of the free reactants to obtain the hydroxide ion–dineopentyl phosphate distance probability distribution and the equilibrium fraction of the non-reactive complex, $\chi_{eq}$; (*ii*) one short simulation for the determination of the diffusion coefficient of the dineopentyl phosphate; (*iii*) a set of 70 simulations in order to reconstruct the reactive and non-reactive complex dissociation kinetics.

## 4. Results and Discussion

As explained in the Theory section, the evaluation of the overall rate constant $k_{H}$ for the P–O cleavage reaction of the $Np_{2}P^{-}$ in water requires the estimate of the rate constants associated with the three reaction steps, described in Scheme (21), using Equation (24).

### 4.1. Evaluation of the Rate Constant $K_{R}$ of the Dineopentyl Phosphate Hydrolysis

The methodology described in the Theory and Methods sections was applied to reconstruct the full kinetics of the hydrolysis of the dineopentyl phosphate. This substrate was selected because of the availability of experimental data [2], as highlighted in the introduction. It is known that the hydrolysis of the dialkyl phosphodiesters with minor steric hindrance around the carbon atoms bound to the oxygen atoms proceeds preferentially via the nucleophilic attack at this carbon atom with respect to the phosphorous atom [17,18] (Figure 2a). However, in the case of the dineopentyl phosphate, the steric hindrance drives the attack onto the phosphorous atom [1], as shown in Figure 2b.

**Figure 3:**
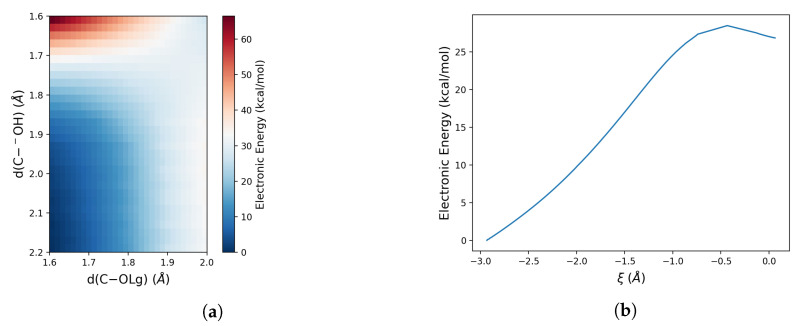
(**a**) Bi-dimensional scan performed along the hydroxide oxygen atom–carbon atom and the carbon atom –leaving group oxygen distances; (**b**) Gas phase electronic energy profile along the reaction coordinate ξ (defined as the difference between the HO${}^{-}$–P and the P–OLg distances).

Therefore, to quantitatively model the free energy profile of the reaction by means of the PMM-MD approach, we first evaluate the minimum energy path along both the P–O and C–O bond cleavages. To study the attack of the hydroxide ion on the phosphorous atom of the dineopentyl phosphate, we performed a TS optimization of the quantum centre and analysis of the TS hessian to confirm that the obtained structure is a saddle point of the potential energy surface of the system. From this TS structure, an intrinsic reaction coordinate calculation was performed to obtain the reaction profile reported in Figure 3b. In the case of C–O bond cleavage, the same calculation on $Np_{2}P^{-}$ failed because of the large steric hindrance due to the neopentyl groups. Therefore, a bi-dimensional PES along the two bond distances involved in the reaction, i.e., the distance between the carbon atom and the oxygen of the hydroxide ($d_{C-OH}$) and between that carbon atom and the oxygen of the leaving group ($d_{C-OLg}$), was performed. These results reported in Figure 3 clearly show a higher energy barrier associated with the C–O bond cleavage (36.1 kcal/mol) with respect to the P–O bond (27.5 kcal/mol). This indicates that the steric hindrance inhibits the C–O bond cleavage. Therefore, we have applied the MD-PMM procedure to evaluate only the thermodynamics and kinetics in solution for the P–O bond cleavage.

**Figure 4:**
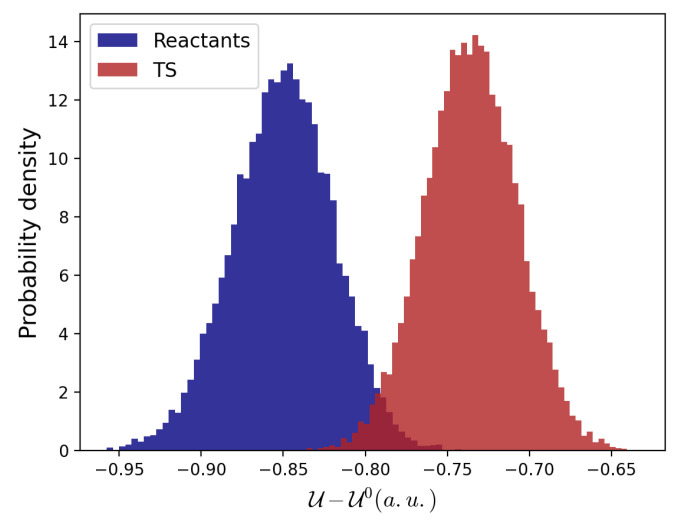
Reactants and TS perturbed electronic energies distributions with respect to their corresponding unperturbed states.

To this end, the electronic energies were evaluated for ∼100 structures taken along the reaction coordinate and perturbed—in the corresponding MD ensemble—by the electric field as provided by the MD simulations. These data indicate that the effect of the environment is to stabilize both the reactants and the TS, as shown by the distributions of the reactant and TS perturbed electronic energies (see Figure 4).

However, the TS perturbed energies decrease less than the reactant state ones, resulting in a higher barrier with respect to the vacuum condition (see Table 2). This effect can be ascribed to a better ability of polar solvent molecules to stabilize two negative charges located on two separated molecular entities compared with a doubly charged molecule.

**Figure 5:**
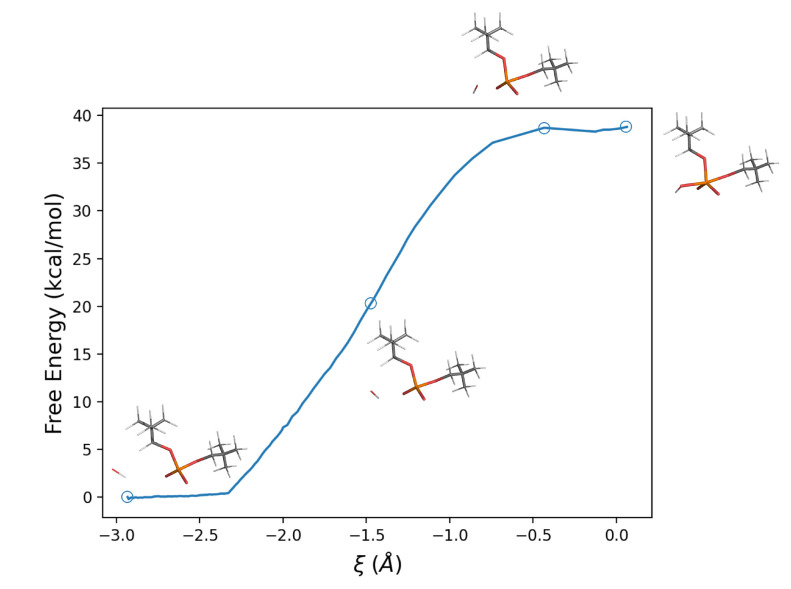
Free energy profile along the reaction coordinate ξ defined as the difference between the HO${}^{-}$–P and the P–OLg distances, for the reaction in aqueous solution.

Using these perturbed energy trajectories in Equation (17), it was possible to calculate the corresponding (Helmoltz) free energy profile along the reaction coordinate, which results in a barrier of 38.8 kcal/mol, Figure 5. Using the statistical–mechanical approach described in the Theory section, i.e., Equation (16), we obtain, for the last (and slowest) reaction step (see Scheme (21)), a kinetic rate constant $K_{R}$ as high as 1.85·$10^{-15}$ s${}^{-1}$.

### 4.2. Evaluation of the Other Kinetic Rate Constants

**Figure 6:**
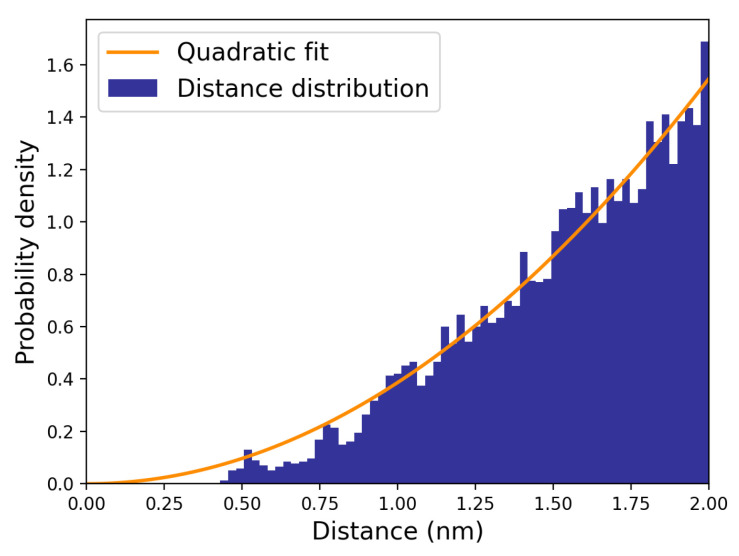
Distribution of hydroxide ion–dineopentyl phosphate distance (navy); quadratic fitting function of the distance distribution (orange).

In order to evaluate the other rate constants for the reaction steps providing the complex dissociation and the non-reactive reactive complex transitions, we computed the overall complex dissociation rate constant ($k_{D}=\chi_{eq}\;k_{2},\;\chi_{eq}=k_{1}/\left(k_{1}+k_{-1}\right)$), assuming pre-equilibrium within the reactant complex, as provided by 70 MD simulations starting from random initial configurations representative of the reactive complex. The direct evaluation of $k_{D}$ can be obtained by monitoring and fitting the overall complex fraction in time, i.e., by first-order modelling the time decay of the trajectory fraction corresponding to the reactants with distances within 1.2 nm. This cut-off value, defining the overall complex radius $r_{0}$, can be identified by inspecting the hydroxide ion–dineopentyl phosphate distance distribution (Figure 6) as provided by MD simulations. When that distribution starts to exhibit a purely quadratic behaviour, the two reactants can be considered to be outside of the non-reactive complex region.

**Figure 7:**
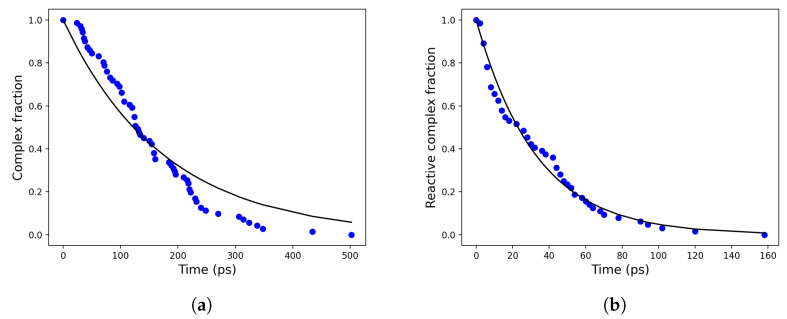
Time decay of the (total) complex (**a**) and the reactive complex (**b**) in blue dots and their corresponding exponential fits in solid black lines.

Analogously, the rate constant $k_{1}$ for the reactive complex → non-reactive complex transition can be computed by monitoring the time decay of the complex fraction corresponding to the reactants with distances within 0.6 nm (i.e., the cut-off radius defining the reactive complex), see Figure 7. The computed values for $k_{D}$ and $k_{1}$ are reported in Table 1.

Moreover, from the 100 ns MD simulation of the free reactants we obtained the equilibrium fraction of the non-reactive complex ($\chi_{eq}$) used to estimate the kinetic constants $k_{-1}$ and $k_{2}$ (see Scheme (21)), also reported in Table 1.

Finally, using all the rate constants for the cleavage reaction steps in Equation (24), it was possible to estimate the overall rate constant $k_{H}=2.61$·$10^{-15}$ s${}^{-1}$ in very good agreement with the experimental data, both reported in Table 1.

Our data clearly show that the first two steps of the reaction have a limited effect on the overall rate of the reaction, as pointed out by the limited difference between $K_{R}$ and $k_{H}$.

### 4.3. Hydrolysis of the Dimethyl Phosphate

In order to confirm that the steric hindrance is able to drive the reaction towards different paths, that is P–O or C–O bond cleavage, the slow step of the hydrolysis reaction of the dimethyl phosphate ($DMP^{-}$) was investigated by means of the same theoretical–computational strategy for both C–O and P–O bond breaks. In agreement with the experimental data, the free energy barrier is remarkably lower for the C–O bond break with respect to the cleavage of the P–O bond.

Assuming that in the case of the $DMP^{-}$ the first two reaction steps exhibit similar behaviours with respect to $Np_{2}P^{-}$, we compare the ratio between the experimental overall rate constants of the two possible bond breaks with the ratio of the corresponding $K_{R}$. From these data, it comes out that our procedure correctly predicts the favourite path (see Table 2).

### 4.4. Mechanistic Considerations

**Figure 8:**
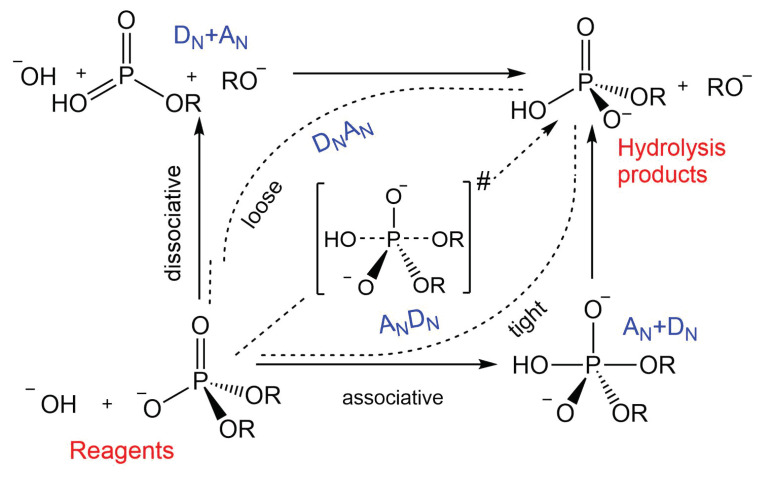
More O’Ferrall–Jencks diagram for the hydrolysis of phosphodiesters. Definition of the associative and dissociative mechanisms.

The hydrolysis of phosphodiesters may proceed via a step-wise mechanism or, alternatively, a concerted pathway (see Figure 8). In the first case, the formation of the intermediate can be generated either by the attack of the nucleophile onto the phosphate unit, i.e., $A_{N}+D_{N}$, mechanism with the generation of a penta-coordinated species, or by the early departure of the leaving group (Lg), named as $D_{N}+A_{N}$ [8,9,41,42]. The latter case can be considered only in the case of substrates provided with very good leaving groups. On the other hand, in a concerted reaction mechanism, the penta-coordinated phosphorane is a transition state instead of an intermediate, namely $A_{N}D_{N}$ pathway, indicated also as $S_{N}2$(P) for its close similarity to carbon nucleophilic substitution. Even in the concerted transformation, the TS can verge to one of the two extreme conditions illustrated above. A transition state that is closer to the associative limit is labelled as $A_{N}D_{N}$, i.e., asynchronous tight. On the contrary, an asynchronous loose $D_{N}A_{N}$ mechanism is closer to the dissociative limit [8,41]. From the analysis of the potential energy surface and the corresponding O${}_{nucleophile}$–P and P–OLg distances (see Figure 3) the reaction pathway does not indicate the existence of any intermediate, as no energy minima is detected along the profile, and therefore the mechanism can be considered concerted and synchronous, or slightly “associative” ($A_{N}D_{N}$). This is in contrast to what has been observed in a previous investigation based on a more approximate theoretical–computational approach and on the implicit treatment of the solvent [9].

## 5. Conclusions

Our theoretical and computational procedure, based on a mixed quantum/classical approach, was employed to investigate a chemical transformation of relevant importance in biological systems. The proposed methodology, based on the perturbed matrix method (PMM), has turned out to be effective in the quantitative prediction of the kinetics and thermodynamics of the hydrolysis of phosphate diesters in water solution. The results obtained by such an approach, in spite of the adopted approximation, are in excellent agreement with the experimental rate constants. Furthermore, this approach provides useful mechanistic insights about the reaction pathway, i.e., C–O bond cleavage vs. O–P bond cleavage for phosphodiesters featuring different steric hindrance conditions, in accordance with the experimental picture. The analysis of the potential energy surface does not show the presence of any energy minimum along the reaction pathway, thus suggesting that the hydroxide-promoted hydrolysis of these compounds proceeds through a concerted “synchronous” mechanism. We believe the present approach can be applied in a wide variety of chemical transformations and therefore can open up the way to a fast and reliable general method to treat bimolecular reactions in condensed phase and in other complex environments.

## Figures and Tables

**Table 1:** Kinetic rate constants of the P–O bond cleavage in $Np_{2}P^{-}$.

Calculated Rate Constants	Value	Dimension
$k_{A}$	0.056±0.009	${\mathrm{ps}}^{-1}\;M^{-1}$
$k_{D}$	0.0056±0.0009	${\mathrm{ps}}^{-1}$
$k_{1}$	0.030±0.002	${\mathrm{ps}}^{-1}$
$k_{-1}$	0.0049±0.0003	${\mathrm{ps}}^{-1}$
$k_{2}$	0.007±0.001	${\mathrm{ps}}^{-1}$
$K_{R}$	$\left(1.85\pm 0.92\right)\cdot 10^{-15}$	$s^{-1}$
$k_{H}$	$\left(2.61\pm 1.23\right)\cdot 10^{-15}$	$s^{-1}\;M^{-1}$
$k_{H}{\left[HO^{-}\right]}^{⊖}$	$\left(2.61\pm 1.23\right)\cdot 10^{-15}$	$s^{-1}$
**Experimental rate constant**		
$k_{H}$	1.4 $\cdot 10^{-15}$	$s^{-1}M^{-1}$
$k_{H}{\left[HO^{-}\right]}^{⊖}$ ${}^{a}$	1.4 $\cdot 10^{-15}$	$s^{-1}$

^*a*^ Hydroxide ion concentration ${\left[HO^{-}\right]}^{⊖}$ = 1 M.

**Table 2:** Comparison of the hydrolysis of $DMP^{-}$ and $Np_{2}P^{-}$. $\Delta U^{0}$ corresponds to the unperturbed electronic energy barrier and ΔA to the free energy barrier.

	${\mathrm{DMP}}^{-}$		${\mathrm{Np}}_{2}P^{-}$	
Bond Cleavage	**P–O**	**C–O**	**P–O**	**C–O**
$\Delta U^{0}$ (kcal/mol)	27.5	28.0	28.5	36.1
ΔA (kcal/mol)	39.5	30.9	38.8	-
$K_{R}$ (s${}^{-1}$)	3.75 $\cdot 10^{-16}$	3.17 $\cdot 10^{-10}$	1.85 $\cdot 10^{-15}$	-
$k_{H}^{exp}$ (s${}^{-1}$M${}^{-1}$)	NA	3.0 $\cdot 10^{-11}$	1.4 $\cdot 10^{-15}$	NA

## Data Availability

Not applicable.

## Supplementary Materials

The following supporting information can be downloaded at: https://www.mdpi.com/article/10.3390/molecules28052152/s1.
