# Supplementary material for: Modelling Complex Bimolecular Reactions in a Condensed Phase: The Case of Phosphodiester Hydrolysis
Source: Molecules. 2023 Feb 24;28(5):2152. doi: 10.3390/molecules28052152 (PMC10004441; doi:10.3390/molecules28052152)
Supplement: Supplementary file 1 [file molecules-28-02152-s001.zip › molecules-2222312-supplementary.pdf]

molecules

MDPI

Supplementary Materials: Modelling Complex Bimolecular Reactions in a Condensed Phase: The Case of Phosphodiester Hydrolysis

1. Transition States Geometries

Coordinates of the transition states.

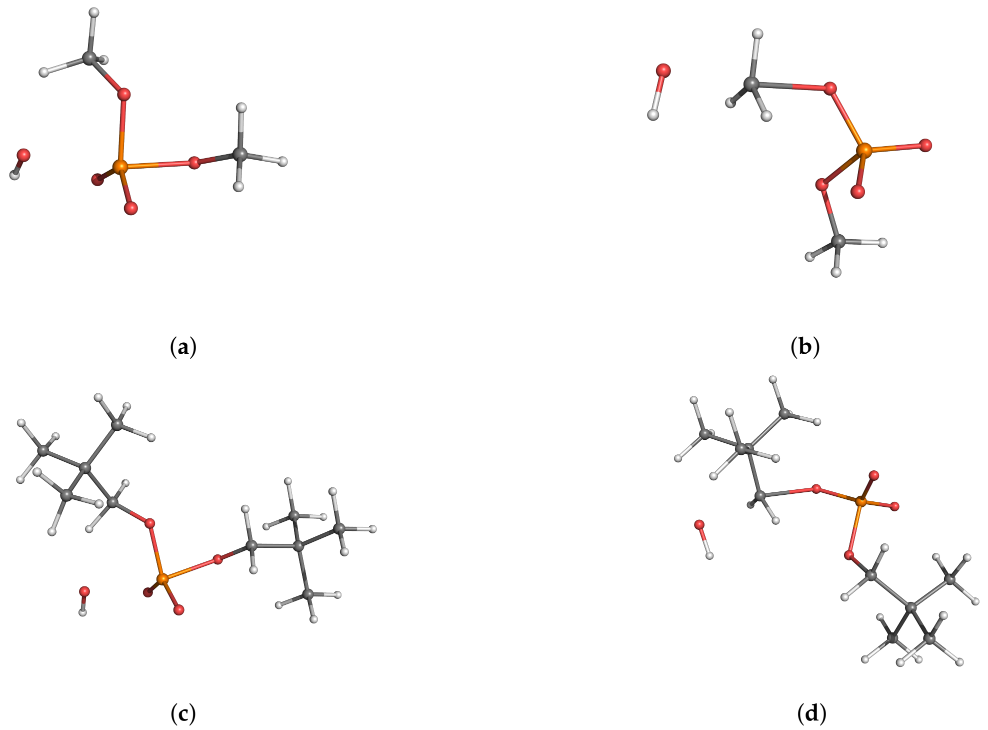

Figure S1. (**a**) TS structure of the DMP^- hydrolysis via the nucleophilic attack on the P atom; (**b**) TS structure of the DMP^- hydrolysis via the nucleophilic attack on the C atom; (**c**) TS structure of the Np_2P^- hydrolysis via the nucleophilic attack on the P atom; (**d**) TS structure of the Np_2P^- hydrolysis via the nucleophilic attack on the C atom.

(a) TS Cartesian coordinates for the DMP^- hydrolysis via the nucleophilic attack on the P atom.

15

P	0.04759421574112	−0.37289143256568	0.16214622555207
O	0.60528130395693	−0.41764604983953	1.56027166244113
O	−0.27373422990218	−1.47125742708887	−0.80475060734840
O	0.06133019988439	1.12633676562971	−0.59017066777321
C	0.93358752866231	2.08300625220335	−0.06780741680389
H	0.87300750397068	2.97402291362842	−0.71299321908439
H	1.95136244274459	1.67523889503383	−0.06785770573723
H	0.65175041929921	2.37744951461538	0.95586318838359
O	−1.63993055450580	−0.03562598869778	0.72571594525318
C	−2.61287534100408	0.00927191918207	−0.24597943999263

H	−2.56052191670488	−0.85874664665831	−0.92335020249146
H	−2.54805223956379	0.91725290955621	−0.88020134053056
H	−3.61280160757574	0.01981769126223	0.23067545442331
O	2.19038204976939	−0.39406106436631	−0.50360843852532
H	2.45204022522786	−0.67867825189471	0.37310656223382

(b) TS Cartesian coordinates for the DMP^{-} hydrolysis via the nucleophilic attack on the C atom.

15

P	−0.752026352730	−0.520874390119	0.086523104568
O	−0.467761894950	−0.202085469628	1.537376447430
O	−1.974728614517	−1.348114862770	−0.226751311029
O	0.503822119798	−0.961453905786	−0.717558509519
C	2.170714968227	−0.161405969195	−0.264537672328
H	1.913378811594	−0.154945178390	0.779168039383
H	2.665517415346	−1.010225355153	−0.694215970735
H	1.951109092432	0.691500008540	−0.876048680786
O	−1.035403394174	1.001777916644	−0.645632422750
C	−2.084222373652	1.713471508742	−0.078455082457
H	−2.169565594618	2.684307158857	−0.590497526226
H	−1.917650472442	1.894104827457	0.993918429378
H	−3.042461554358	1.180066884092	−0.182820404446
O	3.988299695110	0.565952029941	0.089432159668
H	3.776158148935	1.217404796767	0.760499399851

(c) TS Cartesian coordinates for the Np_2P^{-} hydrolysis via the nucleophilic attack on the P atom.

39

P	0.994501106803	−1.508294160479	0.424918839545
O	1.493160066407	−1.746751284499	1.836231538591
O	0.748093738542	−2.473050333501	−0.711057564652
O	1.031949971167	0.093838812166	−0.113374336729
C	1.959347637495	0.979764525536	0.441057772672
H	2.799990397689	0.402847977757	0.847792022831
H	1.480084042791	1.581245552463	1.242340797763
C	2.491729406061	1.934723661459	−0.642129847853
C	3.062742291117	1.104420656270	−1.795441549830
H	3.669720330241	0.278452273872	−1.396022981003
H	3.637535744871	1.743099612381	−2.487571898288
H	2.247171476480	0.623133956455	−2.344518739232
C	3.596556039724	2.786635676870	−0.013123105647
H	4.432491562191	2.150827806806	0.302368601275
H	3.227168247369	3.326572440652	0.871091636057
H	3.980115692939	3.531036647311	−0.727349868236
C	1.366816717597	2.841553583518	−1.149584346813
H	1.711997753369	3.483169143866	−1.975843121955
H	0.987154461961	3.491557389278	−0.347546181953
H	0.535264805115	2.225247762712	−1.507812862710
O	−0.727961405161	−1.255276782671	0.924833838454
C	−1.676469532653	−1.122243639887	−0.065455265363
H	−1.486203896162	−1.833242536148	−0.890771097684
H	−1.672474503717	−0.100754128827	−0.510098549594
C	−3.087964737080	−1.381233017910	0.508785688030

C	−3.354902548824	−0.395767397317	1.647599986622
H	−3.344921641372	0.637399322172	1.274742855749
H	−2.558196573278	−0.486976939534	2.392957990990
H	−4.330787466931	−0.581095704169	2.125026124573
C	−3.141779467029	−2.811434744116	1.048639323956
H	−4.101945549658	−3.020645232825	1.546405500878
H	−2.317587223133	−2.954114728304	1.755005177478
H	−3.005138855744	−3.535342272264	0.235162753403
C	−4.130141237624	−1.196123228441	−0.594447926995
H	−3.942251494653	−1.885827018313	−1.427866634629
H	−4.099460185092	−0.173499915090	−0.994834352507
H	−5.148387891103	−1.383068371680	−0.220201483946
O	3.223294193502	−1.413632394251	−0.135590551798
H	3.449688525783	−1.957152971320	0.625681818549

(d) TS Cartesian coordinates for the Np_2P^- hydrolysis via the nucleophilic attack on the C atom.

39

P	−0.55772502469627	2.34775585747232	0.21680371112853
O	0.52940279284981	3.38760458790239	0.45287211568934
O	−0.71100562738424	1.22424482527671	1.24024869411437
O	−0.11840508602288	1.52734385600618	−1.24566638318436
C	0.59749244070556	0.33500203782766	−1.18394964662555
H	0.18682447107427	−0.34956173491203	−1.95227174873235
H	0.46237051228194	−0.14005609367996	−0.19832476784258
C	2.10854736046578	0.50361718868105	−1.47588244236677
C	2.27825528037575	1.31709421452552	−2.76026946708000
H	1.81160471960984	0.80604938221420	−3.61428549713206
H	3.34237763059918	1.47359640571533	−2.99172217015054
H	1.78664570052166	2.28681977535965	−2.63956037867105
C	2.72286221466578	−0.88802761907775	−1.65368633034191
H	2.27162024785720	−1.41849548210049	−2.50412657881086
H	2.56735959114287	−1.50006681575008	−0.75521449071786
H	3.80584506998383	−0.81948966530378	−1.83208650386095
C	2.79130194440910	1.23084913680704	−0.31444269102237
H	2.27091685803482	2.17347580973399	−0.08930956422940
H	3.84872234404193	1.43142825834984	−0.54999148759089
H	2.75160661303130	0.62021347168598	0.59687022532529
O	−1.91227782208680	2.99403612200267	−0.23670534339572
C	−3.31462570707554	1.92865536542580	−1.09493554929175
H	−2.54214929562294	1.22921288751728	−1.36789602170985
H	−3.45359999070427	2.82141892924291	−1.67901652592318
C	−4.29889164556983	1.59643498592913	0.03328078036807
C	−3.88705645943471	2.18339076529410	1.39194684574128
H	−2.91568554984945	1.79263342509776	1.71017471148298
H	−4.66125609529685	1.91445067427952	2.13086931785157
H	−3.79864701533417	3.27282228829614	1.34145944312284
C	−4.37852469826385	0.06983121333159	0.17719741649429
H	−3.40347063520149	−0.32050109909631	0.49273377803653
H	−4.65619106571069	−0.36619434562403	−0.78770659432806
H	−5.12071416344747	−0.20137594595662	0.94390797976609
C	−5.68199797173199	2.15671379301863	−0.33509184274777
H	−5.64036405130832	3.25347360339039	−0.39268103146735
H	−6.41507222906608	1.88854016496810	0.44120610100794

H	−5.99831637014825	1.76645941858960	−1.30653164917275
O	−4.44417171239319	1.04218388992068	−2.57316496329435
H	−3.73009357529522	0.90941746763293	−3.20658645043508

The reported TS geometries for the DMP^{-} and Np_2P^{-} hydrolysis were obtained, as described in the Method section of the main text, at PBE0 [1]/6-311++G(2d,2p) and 6-31+G(d,p), respectively.

2. Gas-Phase PES of the Nucleophilic Attack at the P Atom in the Np_2P^{-} Hydrolysis

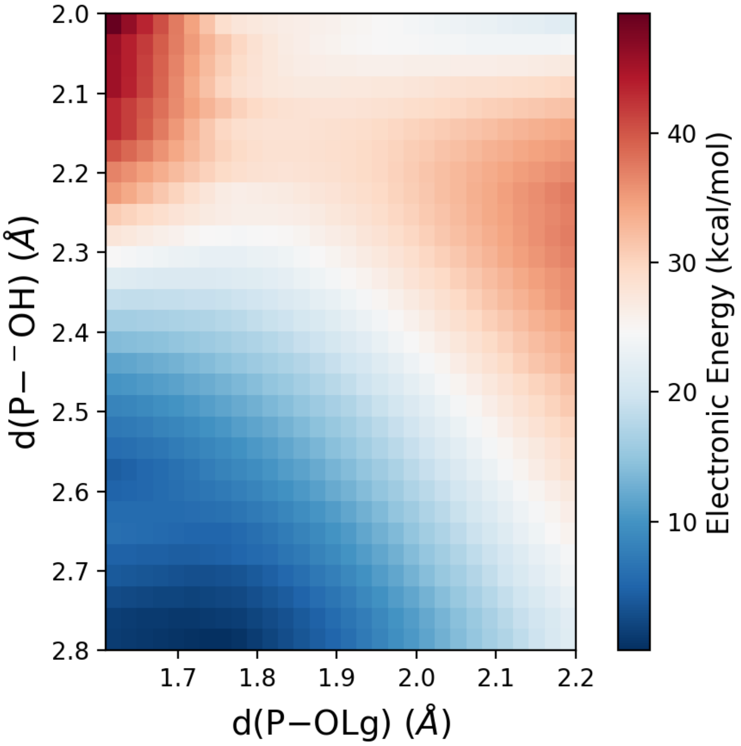

Figure S2. Bi-dimensional scan performed along the hydroxide oxygen atom–phosphorous atom and the phosphorous atom–leaving group oxygen distances.

Reference

1. Adamo, C.; Barone, V. Toward reliable density functional methods without adjustable parameters: The PBE0 model. *J. Chem. Phys.* **1999**, *110*, 6158–6170.
